# Supplementary material for: Identification of photoperiod-induced specific miRNAs in the adrenal glands of Sunite sheep (Ovis aries)
Source: Front Vet Sci. 2022 Jul 22;9:888207. doi: 10.3389/fvets.2022.888207 (PMC9354845; doi:10.3389/fvets.2022.888207)
Supplement: Supplementary file 1 [file Data_Sheet_1.ZIP › Supplementary file/miRDeep2 file.zip!/miRDeep2 file/Know_Structure/SP42A3/oar-mir-29b-1.pdf]

miRBase precursor : oar-mir-29b-1
Total read count : 10597
oar-miR-29b read count : 9670
remaining reads : 927

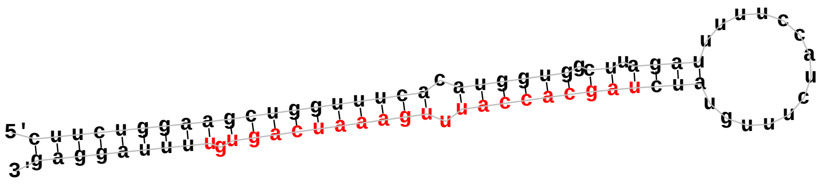

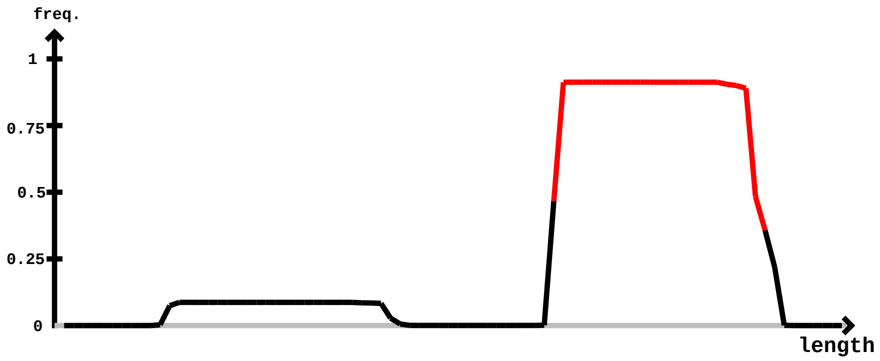

oar-miR-29b

5'- cuucuggaagcugguuucacaugguggcuuagauuuuuccaucuuuguaucuagcaccauuugaaaucaguguuuuaggag -3'	exp		
((((((((((((((((((.((((((.((.((((.............)))))))))))).))))))))))).)))))))))	reads	mm	sample
.........gcugguuucacaugguggcuu...	7	0	seq
.........gcugguuucacaugguggcuuag...	1	0	seq
.........gcugguuucacaugguggcuuaga..	22	0	seq
.........gcugguuucacaugguggcuuagauuuuuc..	1	0	seq
..........cugguuucacaugguggcuu...	11	0	seq
..........cugguuucacaugguggcuua..	7	0	seq
..........cugguuucacaugguggcuuag...	7	0	seq
..........cugguuucacaugguggcuuaga..	513	0	seq
..........cugguuucacaugguggcuuagau...	181	0	seq
..........cugguuucacaugguggcuuagauu..	40	0	seq
..........cugguuucacaugguggcuuagauuu...	2	0	seq
...........ugguuucacaugguggcuu...	1	0	seq
...........ugguuucacaugguggcuuag...	7	0	seq
...........ugguuucacaugguggcuuaga..	55	0	seq
...........ugguuucacaugguggcuuagau...	51	0	seq
...........ugguuucacaugguggcuuagauu..	16	0	seq
.................................uuuuuccaucuuuguauc..............................	2	0	seq
.................................uuuuuccaucuuuguaucG.............................	1	1	seq
..................................uuuuccaucuuuguaucuagcaccauu....................	1	0	seq
....uaucuagcaccauuugaaauca............	1	0	seq
....ucuagcaccauuugaaaucagu..........	9	0	seq
....cuagcaccauuugaaau..............	1	0	seq
....cuagcaccauuugaaauc.............	34	0	seq
....cuaCcaccauuugaaauc.............	1	1	seq
....cuagcaccauuugaaauca............	13	0	seq
....cuagcaccauuugaaaucag...........	20	0	seq
....cuagcaccauuugaaaucGg...........	2	1	seq
....cuagcaccauuugaaaucGgu..........	21	1	seq
....cuagcaccauuugaaaucagu..........	2595	0	seq
....cuagcaccauuugaaaucagug.........	1101	0	seq
....cuagcaccauuugaaaucagugu........	1145	0	seq
....cuagcaccauuugaaaucaguguu.......	6	0	seq
....uagcaccauuugaaauU.............	3	1	seq
....uagcaccauuugaaauc.............	42	0	seq
....uagcaccauuugaaauA.............	1	1	seq

oar-miR-29b

```
cuucuggaagcugguuucacauggugguuagauuuuuccaucuuuguaucuagcaccauuugaaaucaguguuuuaggag
```

```
cuucuggaagcugguuucacaugguggcuuagauuuuuccaucuuuguaucuagcaccauuugaaaucaguguuuuaggag
...................................................uagcaccauuugaaaucG............   22     1     seq
...................................................uagcaccauuugaaauca............   14     0     seq
...................................................uagcaccauuugaaaucag...........   17     0     seq
...................................................uagcaccauuugaaaucGg...........   71     1     seq
...................................................uagcaccauuugaaaucGgu..........   515    1     seq
...................................................uagcaccauuugaaaucagu..........   1161   0     seq
...................................................uagcaccauuugaaaucagug.........   238    0     seq
...................................................uagcaccauuugaaaucagugu........   318    0     seq
...................................................uagcaccauuugaaaucaguguu.......   2304   0     seq
...................................................uagcaccauuugaaaucaguguuu......   9      0     seq
...................................................uagcaccauuugaaaucaguguuuu.....   1      0     seq
....................................................agcaccauuugaaaucagu..........   3      0     seq
....................................................agcaccauuugaaaucaguguu.......   2      0     seq
.....................................................gcaccauuugaaaucaguguu.......   1      0     seq
```
